# Pathogenic role of monocytes/macrophages in large vessel vasculitis

**DOI:** 10.3389/fimmu.2022.859502

**Published:** 2022-07-29

**Authors:** Ryu Watanabe, Motomu Hashimoto

**Affiliations:** Department of Clinical Immunology, Osaka Metropolitan University Graduate School of Medicine, Osaka, Japan

**Keywords:** giant cell arteritis, large vessel vasculitis, macrophages, monocytes, takayasu arteritis, vasculitis

## Abstract

Vasculitis is an autoimmune vascular inflammation with an unknown etiology and causes vessel wall destruction. Depending on the size of the blood vessels, it is classified as large, medium, and small vessel vasculitis. A wide variety of immune cells are involved in the pathogenesis of vasculitis. Among these immune cells, monocytes and macrophages are functionally characterized by their capacity for phagocytosis, antigen presentation, and cytokine/chemokine production. After a long debate, recent technological advances have revealed the cellular origin of tissue macrophages in the vessel wall. Tissue macrophages are mainly derived from embryonic progenitor cells under homeostatic conditions, whereas bone marrow-derived circulating monocytes are recruited under inflammatory conditions, and then differentiate into macrophages in the arterial wall. Such macrophages infiltrate into an otherwise immunoprotected vascular site, digest tissue matrix with abundant proteolytic enzymes, and further recruit inflammatory cells through cytokine/chemokine production. In this way, macrophages amplify the inflammatory cascade and eventually cause tissue destruction. Recent studies have also demonstrated that monocytes/macrophages can be divided into several subpopulations based on the cell surface markers and gene expression. In this review, the subpopulations of circulating monocytes and the ontogeny of tissue macrophages in the artery are discussed. We also update the immunopathology of large vessel vasculitis, with a special focus on giant cell arteritis, and outline how monocytes/macrophages participate in the disease process of vascular inflammation. Finally, we discuss limitations of the current research and provide future research perspectives, particularly in humans. Through these processes, we explore the possibility of therapeutic strategies targeting monocytes/macrophages in vasculitis.

## 1 Introduction

Monocytes are circulating blood leukocytes that play important roles in the inflammatory response, and represent 10% of leukocytes in human blood (1). Monocytes are functionally characterized by their capacity for phagocytosis, antigen presentation, and cytokine/chemokine production, and originate in the bone marrow from a hematopoietic precursor which is common for several subsets of macrophages and dendritic cells (DCs). These cells are not only part of the innate immune system, but also the monocytic lineage that support the activation of the adaptive immune system by antigen presentation (2). Monocytes/macrophages are deeply involved in vascular inflammation including atherosclerosis and vasculitis as well.

**Figure 1:**
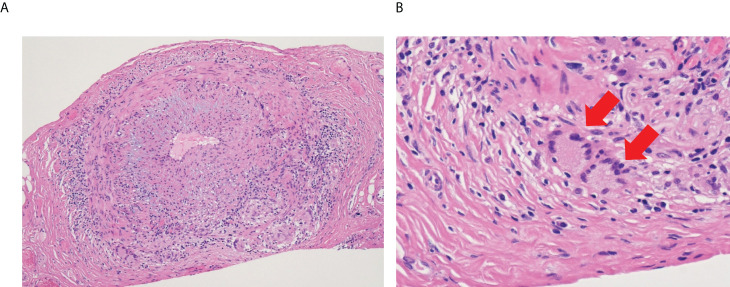
Microscopic image of giant cell arteritis. **(A)** Left temporal artery biopsy from 65-year-old woman with giant cell arteritis (Hematoxylin and eosin staining, x10). Lymphocytes and macrophages form granulomatous inflammation, and intimal hyperplasia causes narrowing of the blood lumen. **(B)** High power field image of the biopsy (Hematoxylin and eosin staining, x40). Red arrows show multinucleated giant cells.

Vasculitis is an autoimmune and/or autoinflammatory vascular inflammation and causes breakdown of the blood vessel walls. Based on the distribution of vessel involvement, it is classified as large, medium, and small vessel vasculitis (3). Large vessel vasculitis affects the aorta and its major branches and include giant cell arteritis (GCA) and Takayasu arteritis (TAK). The hallmark of the two diseases is granulomatous inflammation, which is primarily composed of CD4^+^ T cells and macrophages (4, 5). In GCA, name-giving multinucleated giant cells are often observed in the vascular tissue **(** **Figure 1** **)** and formed by Toll-like receptor (TLR) 2-induced fusion of macrophages (6). Thus, it is obvious that monocytes/macrophages are key players in the pathomechanisms of large vessel vasculitis. Since we have been working on the pathogenesis of GCA, this review will mainly focus on GCA.

The currently available treatments for GCA include glucocorticoids and tocilizumab (TCZ), an IL-6 receptor inhibitor. Even with the adequate use of glucocorticoids, inflammation of the temporal artery remains in about half of the patients after one year (7). Macrophages and giant cells also remain in one in four patients. On the other hand, TCZ reduces vascular inflammation detected by fluorodeoxyglucose-positron emission tomography (8) and flare-up of GCA (9). However, it is difficult to cure the disease, as shown by the flare-up after discontinuation of TCZ in most cases (8). Therefore, clinical unmet needs exist with the current therapies.

This review first summarizes the current knowledge of monocytes/macrophages subsets and the origin of tissue macrophages, particularly in the vascular tissue. Then, the pathogenic roles of monocytes/macrophages in the pathogenesis of large vessel vasculitis are presented. Finally, we discuss future perspectives for therapeutic options targeting monocytes/macrophages in large vessel vasculitis.

## 2 Monocytes/macrophages homeostasis under steady-state and inflammatory conditions

### 2.1 Circulating monocyte subsets

Monocytes differentiated from progenitor cells in the bone marrow reach the circulation. Currently, human circulating monocytes are divided into three subsets based on the expression of superficial CD14 (a cell co-receptor for lipopolysaccharide [LPS]) and CD16 (the low-affinity IgG receptor); “classical” CD14^++^CD16^−^ monocytes (≥90%), “intermediate” CD14^++^CD16^+^ monocytes, and “non-classical” CD14^+^ CD16^++^ monocytes (10). These subsets are characterized by different levels of cell surface markers and chemokine receptors, but there appears to be a developmental relationship between these cells (from classical by intermediate to non-classical) (10). The classical monocytes are involved in a variety of immune response such as inflammation and tissue repair. The intermediate monocytes are characterized by the highest TLR2, TLR4, and human leukocyte antigen-D related expression among monocyte subsets, and have the highest antigen presenting ability. They have superior reactive oxygen species production and have a role in angiogenesis. The non-classical monocytes are called “patrolling” monocytes and have high ability to stimulate CD4^+^ T cells (11). The use of additional markers, such as C-C Chemokine Receptor 2 (CCR2) which is a key mediator of monocyte migration, for better delineation of monocyte subsets has been proposed (12), but its usefulness needs further study.

Conflicting data on cytokine production by the distinct monocyte subsets exist. We have previously reported that the intermediate monocytes treated with LPS produced the most IL-1β, IL-6, and TNFα (13). Wong et al. reported that non-classical monocytes produced the highest IL-1β and TNFα in response to LPS, but that equivalent amounts of IL-6 were secreted by the three subsets. (14). These inconsistencies are probably due to the different isolation methods used to purify the monocyte subsets (11).

An expansion of intermediate monocytes has been implicated in various inflammatory diseases and vascular diseases such as atherosclerosis (15), coronary artery disease (13), and antineutrophil cytoplasmic antibody (ANCA)-associated vasculitis (16). It has been suggested that the ability of intermediate monocytes to present antigens and produce proinflammatory cytokines may be involved in the pathogenesis of such diseases.

### 2.2 Tissue macrophage ontogeny

Tissue macrophages are derived from embryonic or adult hematopoietic stem cell (HSC) progenitor cells under homeostatic conditions (17). Representatives of tissue macrophages are alveolar macrophages (lung), Kupffer cells (liver), osteoclast (bone), microglia (central nervous system), and so on. They are remarkably heterogenous in terms of their surface markers, transcriptome, and epigenomes (18). Monocyte-derived cells also contribute to the macrophage population in the tissues but are mostly associated with a response to inflammatory conditions. It seems more likely that local environmental imprinting is the critical determinant for macrophage identity and function, irrespective of their origin (18).

Recently, the ontogeny of arterial macrophages has been revealed by an elegant method combining the fate-mapping analysis and single-cell RNA sequencing (19). Yolk sac erythro-myeloid progenitors (EMPs) migrate to the arterial adventitia and give rise to adventitial macrophages. Surprisingly, with aging, these adventitial macrophages decline in numbers, and are not replenished by bone marrow-derived monocytes. During vascular inflammation, bone marrow-derived monocytes are recruited to the vascular site and differentiate into adventitial macrophages, while EMP-derived macrophages show self-renewal and contribute to tissue regeneration (19). It has been reported that, during infection, monocytes are educated to be tissue-specific in the bone marrow by signals produced at the site of inflammation (20), but it remains unclear whether this is the case during vascular inflammation.

### 2.3 Macrophage activation and polarization

The most well-described paradigm of macrophage polarization is the M1/M2 polarization axis. M1 and M2 macrophages are also referred to as classically or alternatively activated macrophages, respectively (21). M1 macrophages are activated by the microbial products and proinflammatory cytokines (IFN-γ and/or LPS or TNFα) and characterized by an excess production of proinflammatory cytokines (IL-1β, IL-6, IL-12, IL-23), chemokines, nitric oxide, and reactive oxygen intermediates. In contrast, M2 macrophages are activated by IL-4, IL-10, IL-13, and express mannose receptor (CD206), scavenger receptor A (CD204), and chemokine receptors. High levels of IL-10 are produced by M2 macrophages (22). M2 macrophages are further classified into M2a (IL4/IL-13-induced), M2b (LPS/immune complexes-induced), M2c (IL-10/TGFβ/glucocorticoids-induced), and M2d (tumor-associated factors-induced) macrophages (23, 24).

However, macrophage activation is not that simple. It should be noted that M1 and M2 macrophages are not completely distinct subsets, but they are often overlapping; for example, in atherosclerotic plaque, macrophages expressing both M1 and M2 markers do exist (25). Thus, consensus on how to define macrophage activation *in vitro* and *in vivo* has not yet been fully established. In this context, a group of scientists proposed the updated nomenclature for macrophage activation and polarization (26). In this proposal, they described a set of standards encompassing three principles—the source of macrophages, definition of the activators, and markers to describe macrophage activation—with the goal of unifying experimental standards (26). Technological advances, such as single cell RNA sequencing, may reveal further new macrophage subsets in the future (27).

## 3 Pathogenic role of monocytes/macrophages in large vessel vasculitis

### 3.1 Giant cell arteritis (GCA)

#### 3.1.1 Circulating monocyte population in GCA

An increased number of monocytes (monocytosis) is observed in the peripheral blood of active patients with GCA, and monocyte counts positively correlates with the C-reactive protein (CRP) levels (28). This observation is in line with the report that monocyte-derived macrophages are dominant among tissue macrophages during vascular inflammation (19). Subpopulation analysis using flow cytometry demonstrated that monocytosis in the peripheral blood was attributable to classical monocytes and slightly intermediate monocytes (29). Interestingly, treatment with corticosteroids suppress the numbers of intermediate and non-classical monocytes, but the number of classical monocytes is unaffected (29). It has been reported that glucocorticoid-induced depletion of non-classical monocytes is mediated by caspase-dependent apoptosis (30).

Although monocytes can differentiate into DCs, it has been reported that the number of circulating DCs were comparable between GCA patients and healthy individuals (31). Most quiescent tissues contain resident DC population, but during inflammation, monocyte-derived DCs compensate resident population in the tissue (32). However, it remains elusive whether this is the case in GCA.

#### 3.1.2 Proinflammatory cytokines

It is no doubt that research on monocytes/macrophages in GCA has dramatically progressed since the discovery of IL-6 (33). IL-6 acts on hepatocytes to produce acute phase proteins such as CRP and serum amyloid A (34). It was found that plasma IL-6 levels reflect the disease activity of GCA (35). Although 60–80% of circulating monocytes in patients with GCA can produce IL-6, the major source of IL-6 production was activated macrophages in the vascular lesion (36). Tissue macrophages are activated by IFN-γ released from CD4^+^ T cells (4), and IL-6 shifts naïve CD4^+^ T cell differentiation towards Th17 cells, while inhibiting regulatory T cell (Treg) differentiation (37). Other proinflammatory cytokines, including IL-1β and TNFα, were also localized to tissue macrophages and giant cells (38).

Treatment with corticosteroids diminish IL-1β and IL-6 production from tissue macrophages (39). In contrast, IL-6 receptor inhibitor tocilizumab (TCZ) increases plasma IL-6 levels in patients with GCA (40). TCZ may have little direct effect on suppressing macrophage activation in the vascular tissue and/or block clearance of released IL-6 through IL-6 receptor. However, TCZ reduces relapse and has a steroid-tapering effect on GCA (9) maybe because it restores not only the number of Tregs but also the function of these cells (41–43). Thus, TCZ is widely recommended in the treatment guidelines (44, 45).

IL-12, which is produced by M1 macrophages, is a heterodimeric proinflammatory cytokine that favours the differentiation of Th1 cells (46). Recently, it has been reported that IL-12 promotes conversion from Th17 cells into IFN-γ-producing Th1-like cells, called “non-classic Th1 cells” (47, 48). This transformation is governed by the transcription factor Eomes (49). Indeed, IL-12 is highly enriched in the biopsy-positive temporal arteries (50); therefore, IFN-γ found in the vascular tissues may be derived from Th1 or non-classic Th1 cells. However, ustekinumab, an IL-12 and IL-23 inhibitor, failed to show its efficacy in the treatment of GCA (51).

#### 3.1.3 Proteolytic enzymes and proteinases

Monocytes/macrophages and giant cells not only produce proinflammatory cytokines, but also contribute to tissue destruction. They produce excess proteolytic enzymes and proteinases such as collagenases, cathepsins, and matrix metalloproteinase (MMP)-2 and MMP-9, disrupt external and internal elastic membranes and cause vessel wall destruction (52). Our recent work has revealed that MMP-9-producing macrophages/giant cells are mainly located at the intima-media border and monocyte-derived macrophages from patients with GCA outperformed producing MMP-2 and MMP-9 compared with those from healthy donors (53). Since MMP-2 cleaves the propeptide from the pro-MMP-9 to release enzymatically active MMP-9, this combination of MMPs allows vascular lesions as an active MMP-9-rich environment. We further demonstrated that, using an artificial basement membrane system composed of collagen I and collage IV, MMP-9 released from the circulating monocytes degrades basement membrane and enables CD4^+^ T cell to invade into blood vessel. This study also showed that, using an experimental animal model of vasculitis, blocking MMP-9 was highly effective to protect vascular structure and homeostasis, suggesting that it may serve as a novel therapeutic option for large vessel vasculitis (53).

#### 3.1.4 Colony-stimulating factors

A recent report showed that most of the MMP-9-producing macrophages were CD206 positive and induced by granulocyte macrophage-colony stimulating factor (GM-CSF) (54). Generally, GM-CSF is considered to induce M1 phenotype in macrophages (55, 56), but it may induce M1 plus M2 phenotypes in GCA macrophages. GM-CSF, which is produced by macrophages, T cells, myofibroblasts, and endothelial cells in GCA-affected arteries (57), is expected to be a promising therapeutic target in GCA in recent years. Indeed, treatment of *ex vivo* cultured GCA arteries with the anti-GM-CSF receptor antagonist mavrilimumab successfully ameliorated vascular inflammation through reducing T cell and macrophage infiltration and neoangiogenesis (57). Among T cells, mavrilimumab specifically reduced Th1 cells, but not Th17 cells. In addition, in a phase 2, randomised, double-blind, placebo-controlled trial, mavrilimumab showed superiority to placebo in the analyses of time to flare and sustained remission for patients with GCA (58). Therefore, GM-CSF is not only a macrophage differentiation factor, but is also fundamentally involved in vascular inflammation.

**Figure 2:**
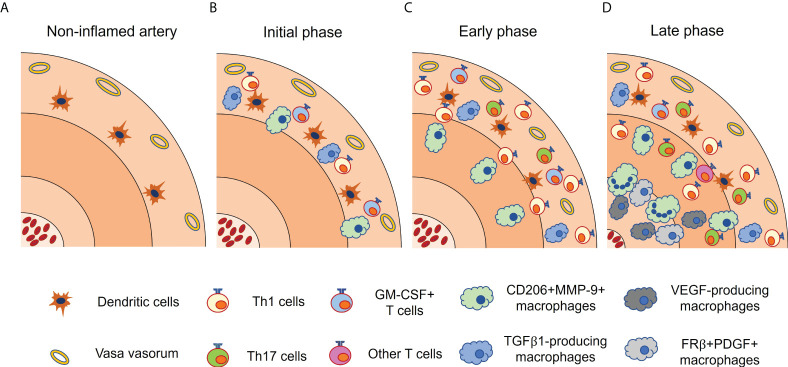
Functionally heterogenous macrophages in giant cell arteritis. Vascular lesion of giant cell arteritis contains a variety of macrophage subsets, each with a characteristic distribution. **(A)** In non-inflamed artery, vascular dendritic cells (vasDCs) reside in the media-adventitial border. **(B)** In the initial phase, vasDCs initiate inflammatory cascade, and recruits T cells and monocytes through chemokines. Infiltrated monocytes are differentiated into CD206+MMP-9+ macrophages by GM-CSF released from activated T cells. TGFβ1-producing macrophages are also present in the adventitia. **(C)** In the early phase, CD206+MMP-9+ macrophages migrate to the media and the media-intima border. Adventitial inflammation is increased. **(D)** In the late phase, CD206+MMP-9+ macrophages often fuse to form multinucleated giant cells and produce M-CSF, which gives rise to FRβ+ PDGF-producing macrophages at the media-intima border. Multiple cytokines and TLR2 are thought to be involved in the formation of multinucleated giant cells. VEGF-producing macrophages are preferentially located in the tunica media and intima. It should be noted that these macrophage subsets are not completely distinct. FRβ, folate receptor β; GM-CSF, granulocyte macrophage-colony stimulating factor; IFN, interferon; M-CSF, macrophage-colony stimulating factor; MMP, matrix metalloproteinase; PDGF, platelet-derived growth factor; TGFβ1, transforming growth factor β1; VEGF, vascular endothelial growth factor.

In contrast, macrophage-colony stimulating factor (M-CSF), which is generally considered to induce M2 phenotype in macrophages (59, 60), is shown to skew macrophages into different phenotypes, namely folate receptor β (FRβ)-positive macrophages (54). M-CSF is mainly produced by CD206^+^/MMP-9^+^ macrophages at the intima-media borders. Collectively, it has been proposed that, at the initial stage of GCA, infiltrated monocytes from the vasa vasorum are primed by local GM-CSF and differentiate into CD206^+^/MMP-9^+^ macrophages. They migrate to media and media-intima junction and promote tissue destruction, while stimulating angiogenesis through IL-13Rα2 signaling (61). At the late stage of GCA, CD206^+^/MMP-9^+^ macrophages often fuse to form multinucleated giant cells and release M-CSF at the intima-media borders. Multiple cytokines (TNF, IL-6, IFN-γ, IL-4, etc) and TLR2 are thought to be involved in the formation of multinucleated giant cells, but the precise mechanism remains unclear (6). M-CSF-skewed FRβ^+^ macrophages produce high concentrations of growth factors that activate myofibroblasts, leading to luminal occlusion (54, 62) (**Figure 2**). Anti-M-CSF antibodies have not been tested in patients with GCA so far.

#### 3.1.5 Growth factors

Tissue macrophages produce growth factors such as transforming growth factor β1 (TGFβ1), platelet-derived growth factors (PDGF), and fibroblast growth factors (FGF) (63, 64). These growth factors are considered to induce an excessive fibroproliferative response leading to luminal occlusion. TGFβ1-expressing macrophages coproduce IL-1β and IL-6 and exhibit a strong preference for localization in the adventitia. Although not clearly proven, given the cytokine profile and localization of TGFβ1-producing macrophages, it is likely that they emerge at the initial disease stage and are activated by IFN-γ released from Th1 cells (62) (**Figure 2**). In contrast, FRβ^+^ macrophages at the media-intima junction emerge at the late disease stage, and produce PDGF, which is closely associated with concentric intimal hyperplasia (54).

In addition, the number of newly formed blood vessel in the adventitia is associated with the production of vascular endothelial growth factor (VEGF), which is localized to tissue macrophages at the media-intima junction (65). VEGF production is augmented by IL-6 (66) and upregulates a NOTCH ligand, Jagged1, on the innermost microvascular endothelial cells. Jagged1 in turn stimulates NOTCH1 receptor on CD4^+^ T cell, skewing CD4^+^ T cell differentiation toward Th1 and Th17 (67). Therefore, anti-VEGF therapy may help to inhibit not only neoangiogenesis but also maldifferentiation of CD4^+^ T cells (68).

#### 3.1.6 Chemokines and chemokine receptors

**Figure 3:**
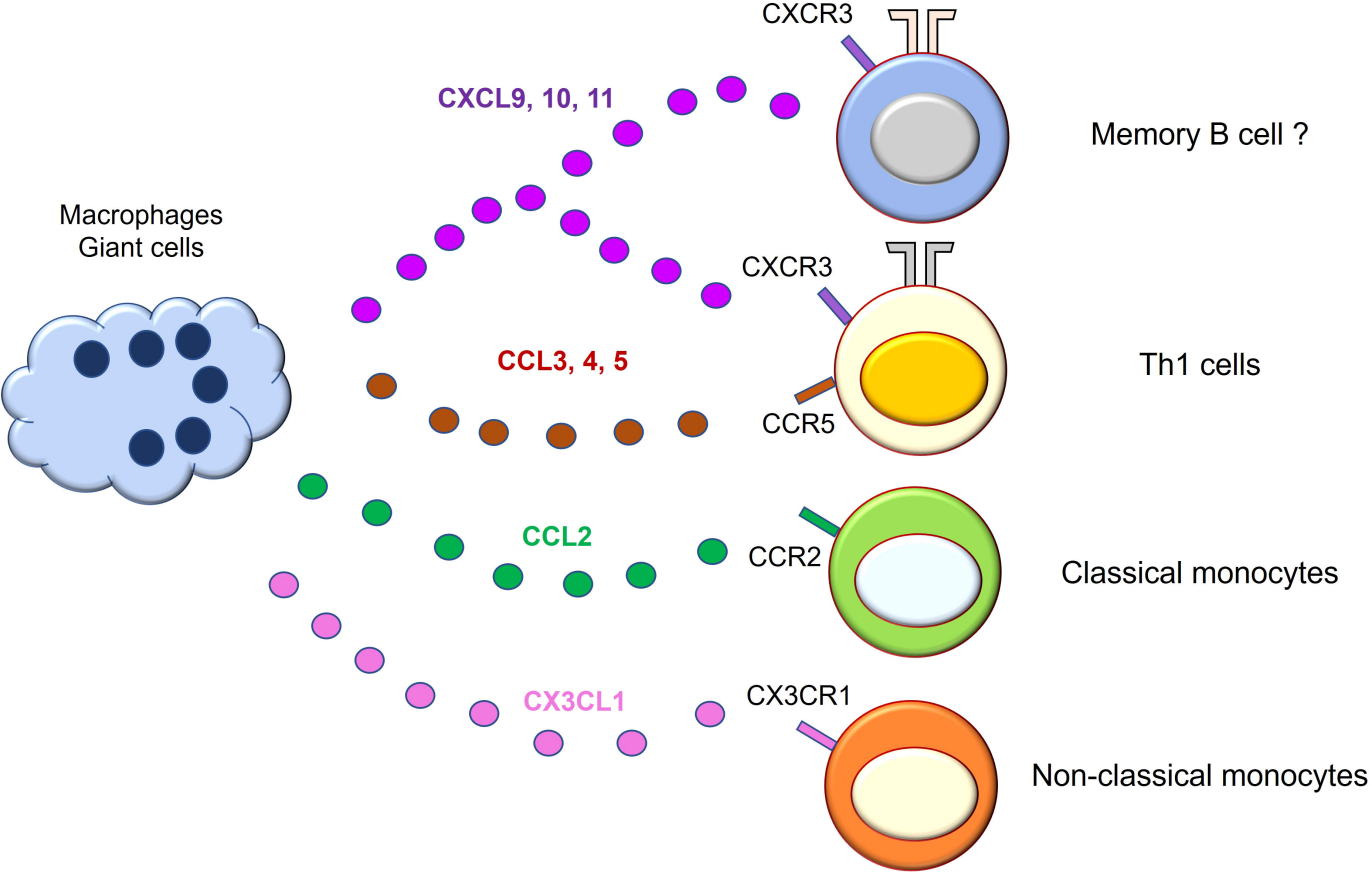
Macrophages/giant cells are professional chemokine producers in giant cell arteritis. Macrophages/giant cells in the vascular lesion of giant cell arteritis actively engage in chemokine production. The released chemokines amplify vascular inflammation by mobilizing cells that express the corresponding chemokine receptors. CXCL9, 10, and 11 recruit Th1 cells and memory B cells through CXCR3 receptor on the cell surface. CCL3, 4, and 5 recruit T cells expressing CCR5. CCL2 recruits CCR2-expressing classical monocytes. CX3CL1 mediates non-classical monocyte mobilization through CX3CR1 receptor. CCL, C-C motif Chemokine Ligand; CXCL, C-X-C motif Chemokine Ligand; CXCR, C-X-C Motif Chemokine Receptor.

Alteration in systemic and local chemokine production and chemokine receptor expression has been reported (**Figure 3**). Among them, C-X-C motif Chemokine Ligand 9 (CXCL9), CXCL10, and CXCL11 levels are elevated in the serum of GCA patients (69). These chemokines are produced by tissue macrophages in response to IFN-γ (70) and recruit Th1 cells through C-X-C Motif Chemokine Receptor 3 (CXCR3). C-C motif Chemokine Ligand 3 (CCL3), CCL4, and CCL5 are also overproduced from macrophages and recruit T cells through CCR5 (71). Not only T cells but also monocytes are recruited by chemokines. For example, classical monocytes are recruited into vascular tissue by the CCL2-CCR2 axis (72), while non-classical monocytes depend on the CX3CL1-CX3CR1 axis (29). These observations suggest that tissue macrophages attract T cells, particularly Th1 cells, and monocytes/macrophages through multiple chemokines, amplifying vascular inflammation. In addition, a recent report has demonstrated that CXCL9 attracts CXCR3^+^ memory B cells, and CXCL13 recruits CXCR5^+^ memory B cells into vascular tissue, respectively (73). Notably, not only tissue macrophages but also vascular DCs produce chemokines such as CCL19 and CCL21, rendering vascular tissue as a chemokine-rich microenvironment (74). Further study is needed to test whether blocking the chemokines and chemokine receptors could have a therapeutic potential.

#### 3.1.7 Costimulatory and coinhibitory ligand expression

Not only costimulatory molecules like CD28, but also coinhibitory molecules, such as programmed death 1 (PD-1), are expressed on T cell surface, and the clinical significance of blocking the PD-1/programmed death ligand 1 (PD-L1) interaction has become clear in cancer immunotherapy. Surprisingly, vascular DCs residing at the media-adventitial boarder have defective PD-L1 expression, which is critically involved in the pathomechanisms of GCA (75). PD-L1-deficient DCs have an increased potential to activate T cells and polarize naïve CD4^+^ T cell differentiation into Th1, Th17, and IL-21-producing T cells (76). Monocytes/macrophages derived from patients with GCA also had decreased expression of PD-L1 (70), although the significance of the deficient expression requires further elucidation. Taken together, PD-L1 immunoinhibitory mechanism to inhibit T cell hyperactivation is defective in myeloid lineage on vascular lesion in GCA. It is necessary to elucidate the mechanism of PD-L1 expression on vascular DCs and tissue macrophages. Also, testing the effect of PD-L1 signal-inducing agents, such as fusion proteins linking the extracellular domain of PD-L1 to the Fc portion of immunoglobulin (PD-L1 Fc), is warranted.

### 3.2 Takayasu arteritis (TAK)

Unlike GCA, it is difficult to perform biopsies of affected lesions in TAK, and only specimens that have undergone surgery are used for research. In addition, large amounts of steroids are often administered prior to surgery, making it rare to obtain an active untreated vascular sample. Thus, the pathogenesis of TAK has not been fully elucidated. Although such bias is undeniable, M1 macrophages are dominant in aortic lesions of TAK (77, 78), which may be linked to excess IFN-γ produced by CD4^+^ T cells, CD8^+^ T cells, and natural killer cells (79). *In vitro* production of MMP-2 and MMP-9 in monocyte-derived macrophages is mildly increased compared with that from healthy donors (80). Steroid treatment transforms M1 macrophages into M2 macrophages and diminishes CCL2-expressing M1 macrophages (78). Thus, M2 macrophages dominate in treated aortic lesion and promote tissue remodeling with an excess fibrotic response.

Recently, single cell RNA sequencing was applied to examine the transcriptome of peripheral blood mononuclear cells from TAK patients (81). The study demonstrated that CD14^+^ monocytes were increased, and gene expressions involved in oxidative stress were enriched. These monocytes may serve as a reservoir of tissue macrophages.

## 4 Discussion

We have reviewed the role of monocytes/macrophages in large vessel vasculitis, particularly in GCA. Of note, functionally distinct macrophage subsets have been increasingly identified in GCA (62), although there were no studies comparing monocytes/macrophages from cranial GCA and those from large vessel GCA. Also, it becomes clearer that circulating monocytes, rather than embryonic progenitor-derived macrophages, cause vascular inflammation by migrating and differentiating into the distinct subsets of macrophages, although it has not yet been fully investigated in human. In particular, since GCA only affects people over the age of 50, the number of tissue resident macrophages in the vasculature may be decreased. Furthermore, low grade inflammation caused by aging, which is called inflammaging, inevitably affects monocyte/macrophages, T cells, and vascular cells both in the circulation and in the vascular tissue (82, 83). Cellular senescence of immune cells is often linked to the senescence-associated secretory phenotype, which could be implicated in the pathomechanisms of GCA (62, 84).

**Figure 4:**
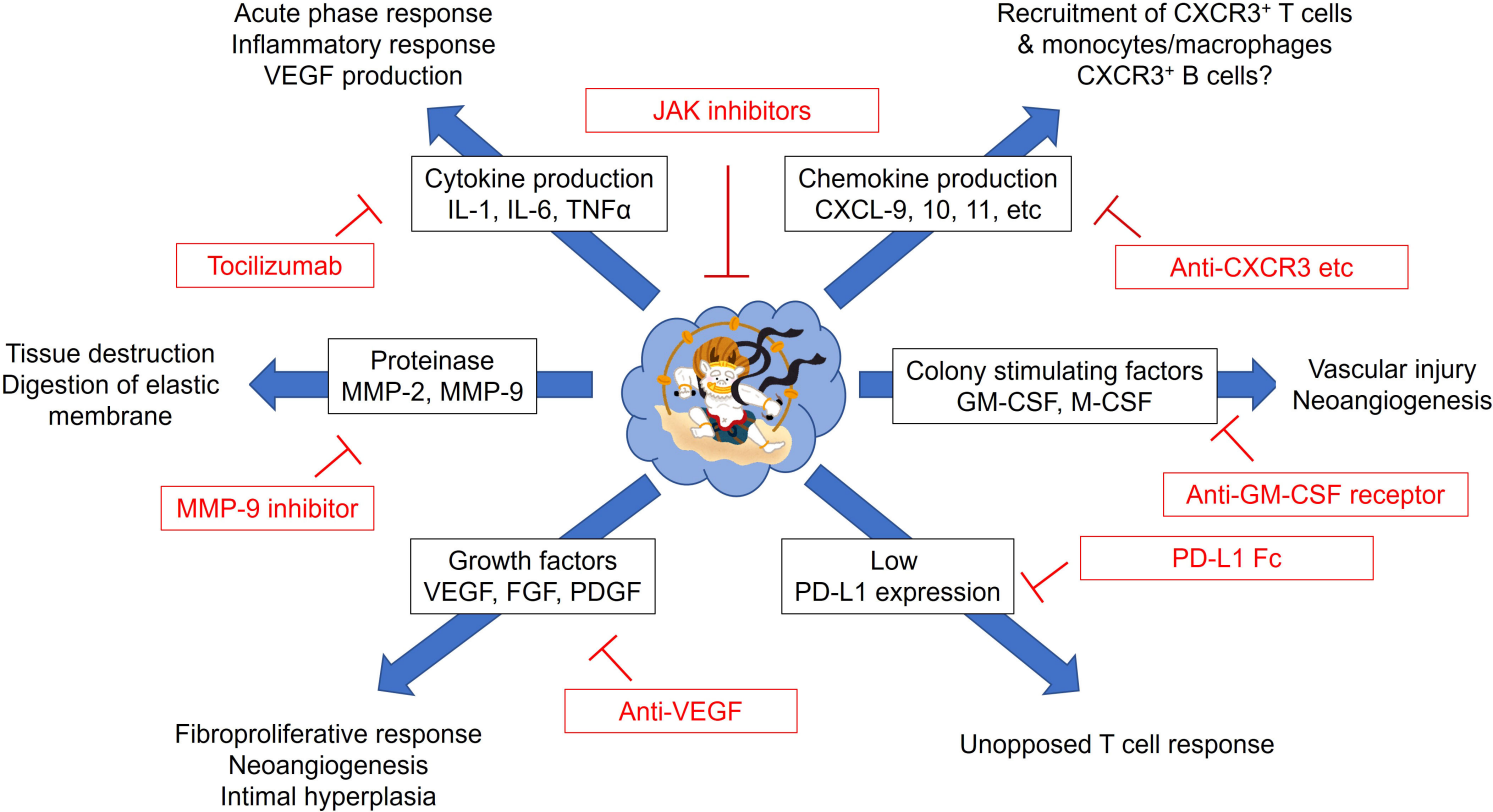
Possible therapeutic strategies for giant cell arteritis targeting monocytes and macrophages. Monocytes/macrophages from patients with giant cell arteritis have pleiotropic functions. Excess production of proinflammatory cytokines (IL-1β, IL-6, and TNFα), chemokines (CXCL9, 10, and 11), proteolytic enzymes (MMP-2 and MMP-9), colony stimulating factors (GM-CSF and M-CSF), growth factors (VEGF, FGF, PDGF) could be targeted by the corresponding inhibitors. Immune dysregulation by defective PD-L1 expression on monocytes/macrophages could be corrected by PD-L1 Fc. Janus kinase (JAK) inhibitors may directly suppress the function of monocytes/macrophages. CXCL, C-X-C motif Chemokine Ligand; CXCR, C-X-C Motif Chemokine Receptor; FGF, fibroblast growth factor; GM-CSF, granulocyte macrophage-colony stimulating factor; M-CSF, macrophage-colony stimulating factor; MMP, matrix metalloproteinase; PDGF, platelet-derived growth factor; PD-L1, programmed death ligand 1; VEGF, vascular endothelial growth factor.

Considering disease mechanisms mediated by monocytes/macrophages in GCA, inhibiting the migration of circulating monocytes or inhibiting their differentiation and function in the tissues may be therapeutic strategies targeting macrophages. The possible therapeutic options in GCA are summarized in **Figure 4**. Blockade of the chemokine and chemokine receptor interaction attracting circulating monocytes and T cells could be the preferential therapeutic option based on the pathomechanisms. However, it is speculated that by the time symptoms appear, a significant number of monocytes have already been recruited to the vascular tissues, and differentiated macrophages are refractory to the current therapies. Therefore, it is unclear to what extent chemokine blockade is effective. In fact, many attempts have been made to treat rheumatic diseases by blocking the chemokine-chemokine receptor interaction, but many of the results have been disappointing so far (85).

Proinflammatory cytokines contribute profoundly to the exacerbation of vasculitis. The GiACTA trial have successfully demonstrated that blocking the IL-6 signal with TCZ suppresses flare of GCA and has steroid-sparing effect (9). However, as mentioned, recurrence after the discontinuation or even during TCZ therapy remains common. In addition, blocking TNFα with infliximab yielded disappointing results for GCA (86). Moreover, anakinra, an IL-1 receptor antagonist, has been shown the efficacy against GCA in case series (87), but its efficacy and safety have not fully been confirmed in the large-scale trials. Therefore, accumulating evidence shows that single cytokine inhibition may not be sufficient to completely diminish vascular inflammation.

As we have seen, treatment that suppresses a single therapeutic target, such as chemokines, proinflammatory cytokines, proteolytic enzymes, or growth factors, may not be sufficient for treating GCA. A combination of these or agents that inhibits the multiple cellular signaling, such as Janus kinase (JAK) inhibitors, may be effective (88). Multiple cytokines which are implicated in the pathomechanisms of GCA, such as IL-6, IFN-γ, IFN-α, GM-CSF, utilize the JAK-signal transducer and activator of transcription (STAT) pathway (89). Indeed, increased activities of the JAK-STAT pathway has been reported both in the vascular lesions and in circulating T cells (90, 91). In experimental animal model of large vessel vasculitis, JAK inhibitors not only reduced T cell infiltration and T cell-derived cytokine production, but also inhibited macrophage infiltration and growth factor production, resulting in reduced neoangiogenesis and intimal hyperplasia (90).

CD4^+^ T cells from patients with TAK are also dependent on the JAK-STAT pathway (92). In addition, genome-wide association study has demonstrated that *IL-12B* is an susceptibility gene in TAK (93) and risk allele of *IL-12B* was associated with vascular damage in TAK (94). Since IL-12 utilizes the JAK-STAT pathway as a downstream signaling, JAK inhibitors could be promising agents for TAK as well (92, 95).

Finally, PD-L1 deficiency seems not specific to GCA monocytes. Monocytes derived from ANCA-associated vasculitis have the same defect (96). Lower PD-L1 expression leads to increased stimulatory capacity of monocytes, thus leading to overactivation of CD4^+^ T cells. The defective PD-L1 expression was due to an enhanced lysosomal degradation of PD-L1 (96). As the efficacy of PD-L1 Fc has been shown in a mouse model of lupus (97), PD-L1 Fc may induce negative signals to overactivated T cells in vasculitis and ameliorate vascular inflammation. Alternatively, agents that inhibit PD-L1 degradation in lysosomes may have therapeutic potentials.

In conclusion, recent advances in the research have clarified the origin and various roles of monocytes/macrophages in vasculitis. Drugs that inhibit multiple therapeutic targets simultaneously, rather than a single target, or agents that block multiple cellular signaling may be effective; however, verification of the efficacy and the safety of such drugs is essential.

## Author contributions

RW drafted the manuscript. MH revised and finalized the manuscript. All authors contributed to the article and approved the submitted version.

## Funding

This work was in part supported by JSPS KAKENHI Grant Number 20K17418, a grant-in-aid of the Cardiovascular Research Fund, Tokyo, Japan and grant for Promoting Research and Survey in Rheumatic Diseases by Japan Rheumatism Foundation to RW.

## Acknowledgments

We would like to thank Dr. Yuto Kaimi (Department of Pathology, Osaka Metropolitan University Graduate School of Medicine) for pathological analysis and Enago for the language review (https://www.enago.jp/).

## Conflict of interest

The authors declare that this review does not contain any commercial or financial relationships that could be construed as a potential conflict of interest.

## Publisher’s note

All claims expressed in this article are solely those of the authors and do not necessarily represent those of their affiliated organizations, or those of the publisher, the editors and the reviewers. Any product that may be evaluated in this article, or claim that may be made by its manufacturer, is not guaranteed or endorsed by the publisher.
